# Tsetse fly inspired steerable bone drill—a proof of concept

**DOI:** 10.3389/fbioe.2023.1197940

**Published:** 2023-06-07

**Authors:** Esther P. de Kater, Rob Müller, Aimée Sakes, Paul Breedveld

**Affiliations:** Bio-Inspired Technology Group, Faculty of Mechanical, Maritime, and Materials Engineering, Department of BioMechanical Engineering, Delft University of Technology, Delft, Netherlands

**Keywords:** bio-inspired design (BID), biomimetic, medical device design, orthopaedic surgery, bone drill, steerable drill

## Abstract

The fixation strength of pedicle screws could be increased by fixating along the much stronger cortical bone layer, which is not possible with the current rigid and straight bone drills. Inspired by the tsetse fly, a single-plane steerable bone drill was developed. The drill has a flexible transmission using two stacked leaf springs such that the drill is flexible in one plane and can drill along the cortical bone layer utilizing wall guidance. A proof-of-principle experiment was performed which showed that the Tsetse Drill was able to successfully drill through 5, 10 and 15 PCF cancellous bone phantom which has similar mechanical properties to severe osteoporotic, osteoporotic and healthy cancellous bone. Furthermore, the Tsetse Drill was able to successfully steer and drill along the cortical wall utilizing wall guidance for an insertion angle of 5°, 10° and 15°. The experiments conclude that the tsetse fly-inspired drilling method is successful and even allows the drilling along the cortical bone layer. The Tsetse Drill can create curved tunnels utilizing wall guidance which could increase the fixation strength of bone anchors and limit the risk of cortical breach and damage to surrounding anatomy.

## 1 Introduction

### 1.1 Bone drilling

**FIGURE 1:**
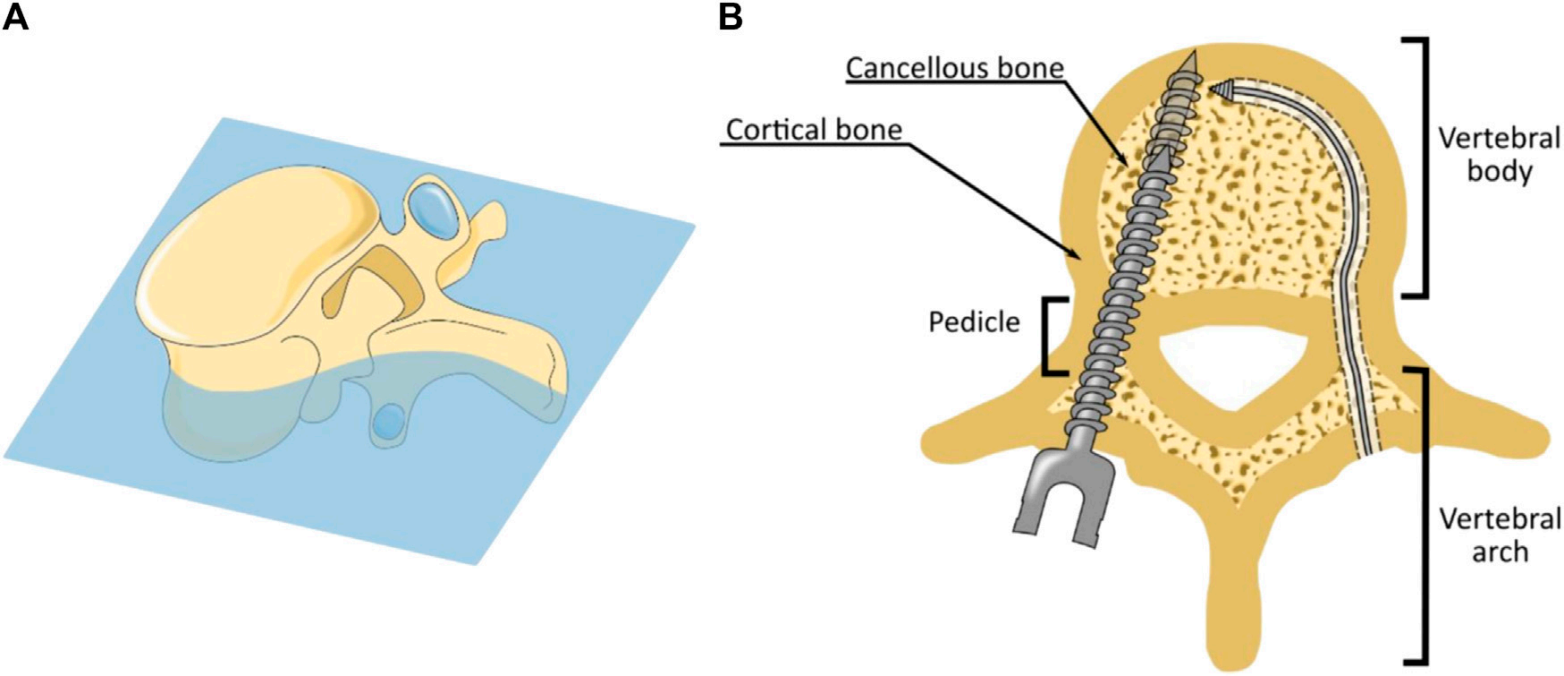
Spinal Fusion Surgery. **(A)** Cross-section of a lumbar vertebra. **(B)** During spinal fusion surgery pedicle screws are placed through the pedicles (left). The fixation strength of the pedicle screw can be increased using bi-cortical fixation in which the distal tip of the pedicle screw is embedded in the anterior cortical bone layer (left). Drilling of a curved tunnel along the cortical bone layer could further increase the fixation strength of spinal bone anchors (right).

Orthopaedic surgery concentrates on the fusion, fixation and reshaping of bones using bone drills, saws and screws. An example of orthopaedic surgery is spinal fusion (Figure 1). This surgical procedure accounted for 14,1 billion dollars in aggregate costs in 2018 in the US alone, which is more than any other procedure that year in the US (McDermott and Liang, 2006). In spinal fusion surgery, adjacent vertebrae are fused in the correct position using pedicle screws and rods. Fusion is achieved by creating a tunnel that runs through the pedicles into the vertebra body using an awl. A pedicle screw is placed in this pre-made tunnel to provide the required fixation (Figure 1B). The success rate of the spinal fusion greatly depends on the fixation strength of the pedicle screws within the bone. Insufficient fixation of the pedicle screw can result in screw loosening, which prevents the desired fusion (Zindrick et al., 1986).

The study conducted by Wu *et al.* (Wu et al., 2011) reported screw loosening in 4.7% of the placed pedicle screws in spinal fusion surgery. A major cause of screw loosening is related to vertebral anatomy. Vertebrae consist of a thin but compact and strong outer layer of cortical bone, which encloses the much softer and porous cancellous bone. The fixation strength of pedicle screws mainly results from contact with the compact cortical bone inside the pedicle. However, the majority of the pedicle screw is surrounded by the softer cancellous bone. Especially for patients suffering from osteoporosis, the fixation strength of pedicle screws is limited due to the decrease of bone density of the cancellous bone (Burval et al., 2007).

The fixation strength of pedicle screws can be increased using bi-cortical screw fixation in which the insertion path is chosen such that the distal end of the pedicle screw is placed in the cortical bone at the anterior side of the vertebral body (Figure 1B). As a result, the pedicle screw has contact with the cortical bone layer in the pedicle and at the distal end of the pedicle screw, which increases the pull-out strength (Zindrick et al., 1986), (Cloutier et al., 2007). Following this principle, a curved tunnel along the cortical wall through which the bone anchor can be placed could further increase the fixation strength of the bone anchor due to the increased contact with the cortical bone layer (Figure 1B). In addition to the enhanced fixation strength achieved by drilling along the cortical bone layer, the curved path itself can further increase the fixation strength of spinal bone anchors by utilising this macro-shape grip with the bone. However, in order to create a curved tunnel along the cortical bone layer, a steerable bone drill is required. Unfortunately, as of today, there is no steerable bone drill clinically available (Sendrowicz et al., 2019).

### 1.2 State-of-the-art: steerable bone drilling

The development of a steerable bone drill is challenging due to the interplay between the need for low bending stiffness to follow a curved trajectory with the need for high axial stiffness that is necessary to drill through bone. Furthermore, the drilled tunnel should remain fully within the cortical bone layer to avoid damage to the surrounding anatomy, which is challenging due to the lack of imaging modalities that can visualize the drill path in real-time. Additionally, the relatively small diameter of the pedicles restricts the outer diameter of the drill to 4 mm, as this is the smallest pedicle diameter measured, which complicates manufacturing (Zindrick et al., 1987).

**FIGURE 2:**
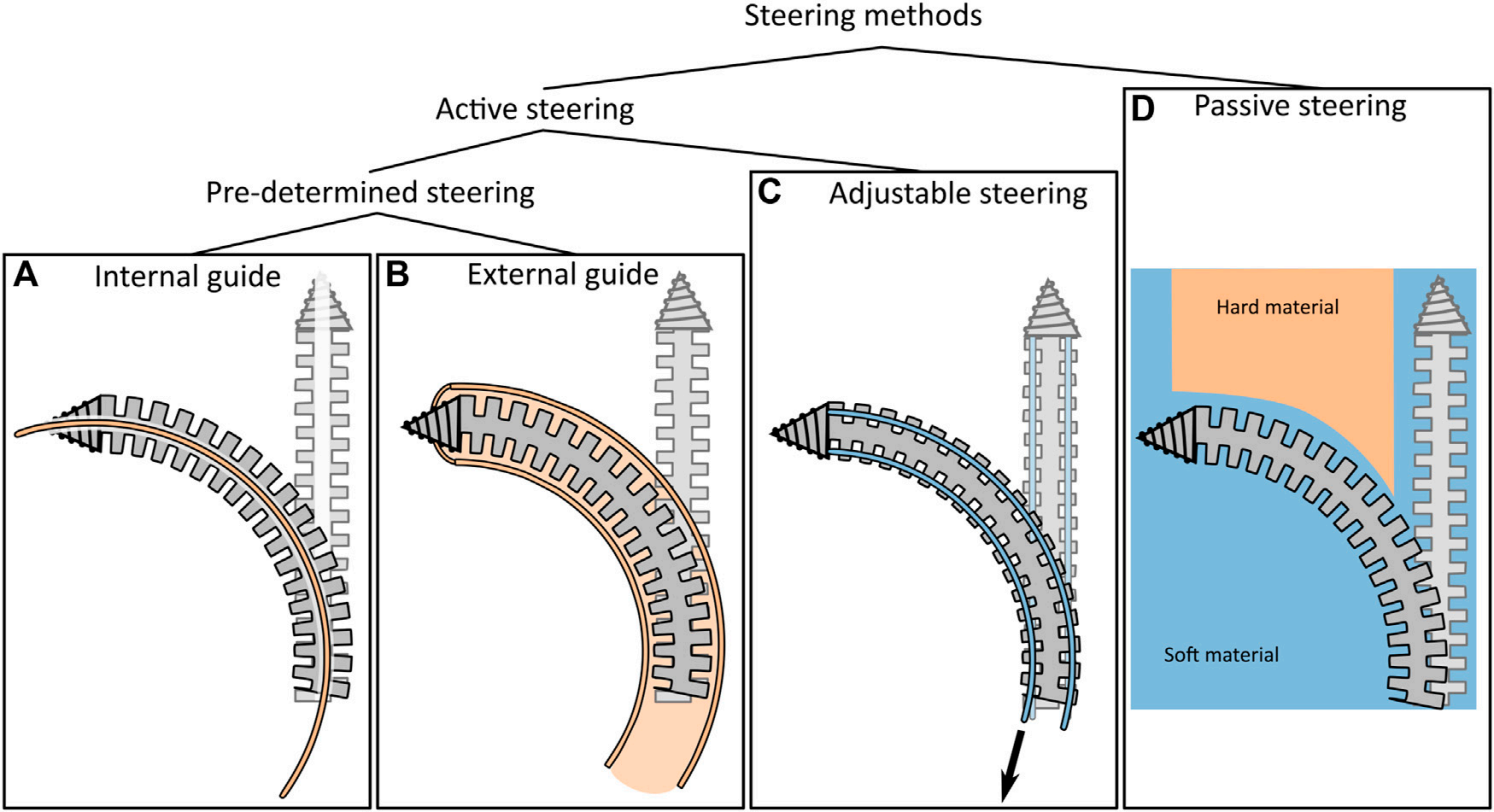
Overview of steering techniques for bone drills. Within the category “Active steering” a distinction between “Pre-determined steering” and “Adjustable steering” is made. **(A)** Use of an internal guide to actively bend the drill. **(B)** Use of an external guide to actively bend the drill. **(C)** Adjustable steering methods including the use of tendons. **(D)** Use of passive steering to passively bend the drill using the environment.

Currently, there are no commercially available bone drills that can drill curved tunnels through bone and allow for real-time path adaptation. However, flexible reamers in Anterior Cruciate Ligament (ACL) reconstruction in the knee, are commercially available. These devices are utilized to create a pre-determined curved tunnel in the femur, through which a ligament graft can be passed to reconstruct the ACL (Forsythe et al., 2017). Flexible reamers use a bendable but torsion-stiff structure to axially rotate the tip while still allowing for bending motions. Although the reamers can be bent, they do not allow for real-time path adaptation as they are used in combination with an internal or external pre-curved guide (Figures 2A, B) (Stryker, 2016; Stryker, 2019; Lenkbar, 2022; Zimmerbiomet, 2022). Drilling along the cortical bone layer in vertebrae with these types of instruments would thus require patient-specific guides that can be used inside the vertebra and need to be manufactured pre-operatively.

The scientific literature describes a variety of steerable bone drill designs that are actively steerable. The proposed drills share many similarities with commercially available reamers, as most drills also use a flexible drive shaft to rotate the drill tip while allowing for the bending of the drill (Watanabe et al., 2011; Alambeigi et al., 2017; Ahmad Fuad et al., 2018; Ma et al., 2021; Wang et al., 2022). Wang *et al.* (Wang et al., 2022) describe a rigid bone drill containing a tip that can deflect using a tendon-driven joint (Figure 2C) to target a larger lesion area through a single entry point. However, the drill is not able to drill a curved tunnel due to the rigid design of the shaft. Ahmad Faud *et al.* (Ahmad Fuad et al., 2018) describe a flexible bone drill that consists of multiple interconnected rigid segments. The drill is intended for use in total hip arthroplasty to create a curved cavity in the femur. Alambeigi *et al.* (Alambeigi et al., 2017) and Ma *et al.* (Ma et al., 2021) both describe a steerable bone drill that comprises a bendable but axially stiff structure, utilizing compliant joints. The drill tip can deflect by tensioning internal steering cables. Although these drills can in principle drill curved tunnels by selectively pulling the steering cables, the required steering forces are relatively high due to the interaction forces with the surrounding bone when deflecting the tip of the drill. Watanabe *et al.* (Watanabe et al., 2011) try to reduce the required tension forces by making the bendable section more compliant at the tip of the drill. Using this steering principle leads to cable tensioning forces of over 30 N required to generate a drill tip deflection of 20°.

Besides the drill designs presented in scientific literature, the review of Sendrowicz *et al.* (Sendrowicz et al., 2019) shows that in the patent literature, many designs for steerable bone drills are proposed. One thing that must be noted is that the majority of these patents (78%) describe drills that are only able to make pre-defined curved tunnels (Figures 2A, B). The path can thus not be changed during the intervention. As a result, exact information on the patient’s anatomy is required to define the path pre-operatively and the drill path cannot be adapted to deformation or displacements that may occur during the procedure. In the overview of Sendrowicz *et al.* (Sendrowicz et al., 2019)*,* only the patent of Bonutti (Bonutti, 2003) describes a drill design that can drill a multi-curved path that is adjustable during insertion (Figure 2C). The patent describes a drill containing inflatable elements in its shaft that allow for deflection of the tip. However, to our knowledge, the design has not been manufactured or tested in a close to clinical setting.

### 1.3 Challenges in steerable bone drilling

Conventional drills use an axially rotational motion to advance through the target material. To make such a drill flexible, the drill must be flexible in two orthogonal bending planes due to the rotational motion of the shaft. However, for drilling a continuous tunnel along the cortical bone layer of the vertebral body, planar bending in a single plane would in theory be sufficient, as indicated in Figure 1. The drill would only need to be flexible in one bending plane while it could be completely rigid in the orthogonal plane, which would eliminate buckling in this plane and could increase the buckling resistance of the drill overall. Furthermore, such a planar bending drill would open pathways for the application of non-rotational drilling methods such as hammering or milling, to achieve the curved pathway in the vertebra.

Steering of a drill can be achieved using internal forces applied on the drill that are initiated by the user, as explained in Section 1.2. This includes using steering cables or the use of a pre-curved internal or external guide (Figures 2A–C). The internal forces that are required to bend a drill tip enclosed in bone tissue are often rather high due to the material properties of cancellous bone. This puts high strains on the drill and could in extreme cases cause mechanical failure. An alternative to active steering of the drill would be to passively steer the drill using external forces that are exerted on the drill by the environment (Figure 2D). Cortical bone is compact and much stronger than porous cancellous bone. Therefore, drilling through the cortical bone will result in higher cutting forces. These cutting forces could in theory be used to deflect the drill tip such that the drill will take the path of the least resistance and steer along the cortical bone layer. We will refer to this type of steering as “wall guidance”.

**FIGURE 3:**
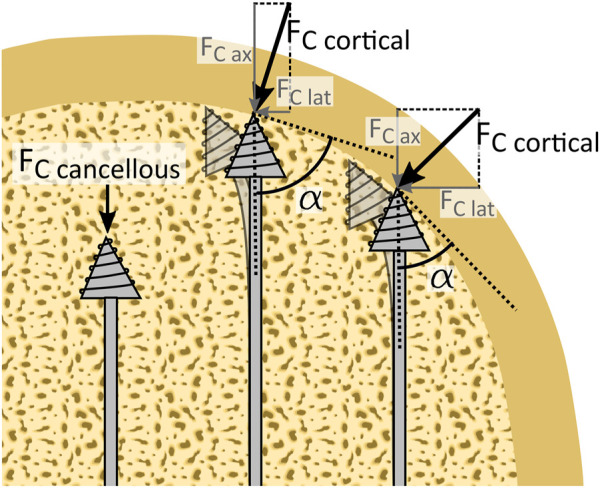
Schematic representation of wall guidance steering. Wall guidance allows the drill to passively steer along the cortical bone layer. When the drill is surrounded by cancellous bone, an axial cutting force ( $F_{C\;cancellous}$ ) acts on the drill tip. When the drill tip is in contact with the significantly harder cortical bone the cutting force ( $F_{C\;cortical})$ increases. The orientation of the cutting force depends on the impact angle α between the drill and the cortical bone layer.

Wall guidance is based on the drill taking the path of least resistance. When drilling through the softer and porous cancellous bone, the cutting force induced by the cancellous bone ( $\left.F_{C\;cancellous}\right)$ is axial and will not cause a deflection, except when the cutting force exceeds the buckling strength of the drill (Figure 3). The cutting force will drastically increase when the drill tip comes into contact with the much stronger cortical bone ( $F_{C\;cortical}$ ). Depending on the impact angle ( α ) between the drill and the cortical bone, a lateral force ( $F_{C\;lat}$ ) will be introduced, which can be used to deflect the drill (Figure 3). This way wall guidance can be used to drill along the cortical bone wall without the need for active steering by the user. However, it should be noted that in vertebrae there is no clear transition point from cancellous to cortical bone. Rather there is an approximately 2 mm thick transition zone where the porous cancellous bone converges in the compact cortical bone (Zebaze et al., 2013). This gradual change in bone density and thus in drilling resistance might complicate steering by wall guidance. Even so, we expect only a small effect due to the low thickness of the transition zone in most vertebrae.

### 1.4 Goal of this study

The goal of this study is to develop a planar steerable bone drill for use in spinal fusion surgery that can drill along the cortical bone layer to increase the fixation strength of a spinal bone anchor. We explore wall guidance as a method to drill along the cortical bone layer without the need for active steering by the user.

## 2 Development of the Tsetse Drill

### 2.1 Bio-inspiration: Tsetse fly proboscis

Insects can drill through relatively hard materials with their slender ovipositors or mouthpieces. These ovipositors or mouthpieces are often flexible or even steerable during insertion (Cerkvenik et al., 2019). This makes insects an interesting inspiration source for the design of a novel, steerable bone drill. Especially the drilling method utilised by the tsetse fly to cut through the host’s skin is of interest as the rotational motion that is used to cut the skin, occurs around an axis perpendicular to the linear advancement of the proboscis. This makes the drilling mechanism bendable in one plane and thus an interesting inspiration for the development of a planar steerable drill.

**FIGURE 4:**
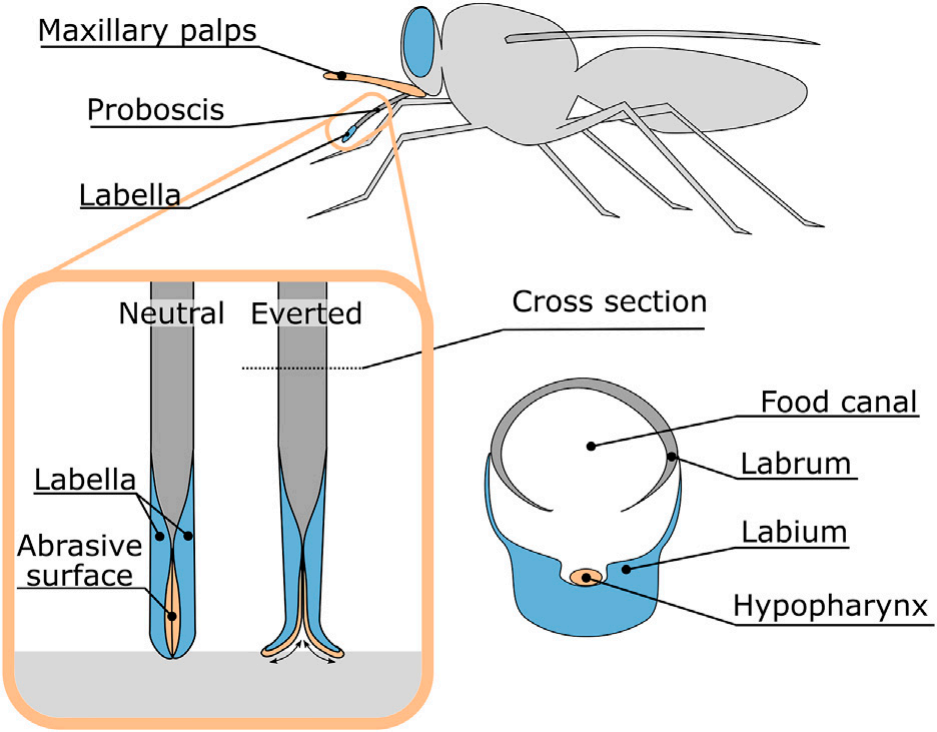
Anatomy of the tsetse fly proboscis and the rasping motion of the proboscis during feeding.

The tsetse fly proboscis, a tubular organ used to suck blood, consists of two mouth parts; the labrum and the labium which form the food canal through which the blood is transported (Figure 4). The labium is located ventrally from the hypopharynx, a tubular organ through which saliva is transported. The thicker tip of the labium, which is known as the labella, surrounds both the hypopharynx and the labrum and is used to cut through the skin of the host to subsequently feed on the blood of the host. For this purpose, the labella are covered with sharp teeth. Normally the teeth are located within the proboscis, but during feeding, the teeth are repeatedly everted and inverted using a combination of muscle forces and haemostatic pressure to create a rasping motion to cut through the skin (Krenn and Aspöck, 2012), (Jobling, 1933). As a result, the proboscis is repetitively pulled into and pushed out of the tissue. A pulling force on the distal tip, to pull the proboscis deeper into the substrate, has a major advantage over pushing the proboscis forward at the proximal end, as it limits the risk of buckling of the proboscis.

### 2.2 Drill tip design

**FIGURE 5:**
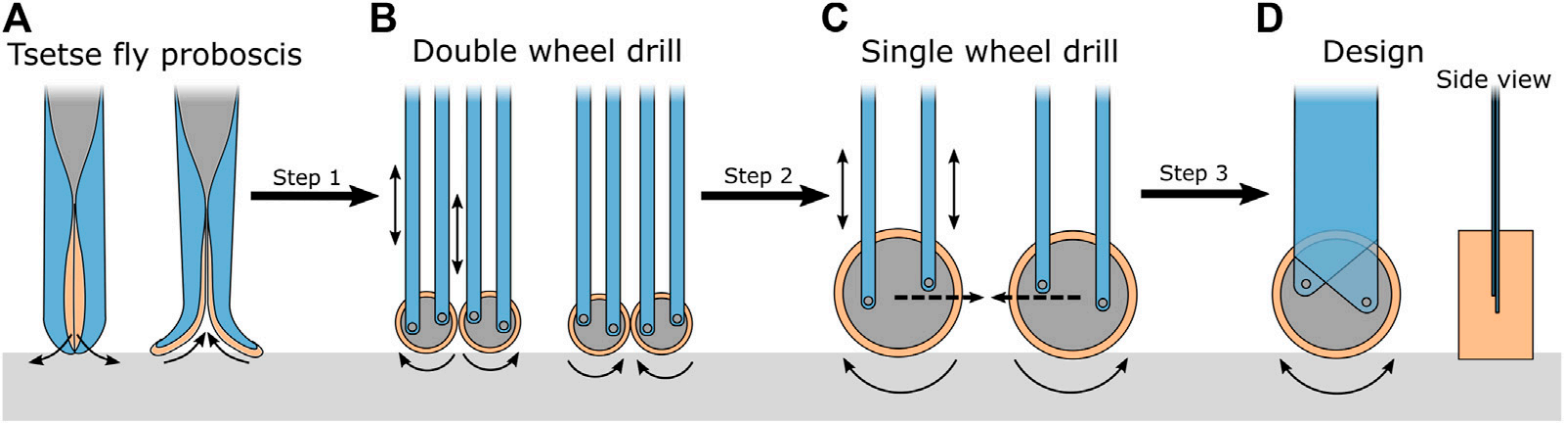
Schematic representation of the proboscis of the tsetse fly and the transformation to the drill tip design. **(A)** Schematic illustration of the tsetse fly proboscis. **(B)** Double wheel design of the bone drill. **(C)** Single wheel design of the bone drill. **(D)** Final conceptual design of the bone drill. The motions of the parts are indicated with arrows. The “walking” behaviour of the drill tip is indicated with the dotted arrow.

The drilling method of the tsetse fly proboscis shows potential for use in an planar flexible bone drill. However, the large number of independently moving and flexible parts are difficult to manufacture with conventional machining techniques and to actuate reliably. The drilling method used by the tsetse fly is, therefore, simplified such that it can be implemented in the design of the steerable bone drill (Figure 5A). The motion of the labella during drilling could be simplified as an oscillating rotation of two wheels in opposing directions (Figure 5B). The advantage of two oppositely oscillatory rotating wheels is that the lateral forces acting during drilling are counteracted. As a result, the proboscis is repetitively pulled into and pushed out of the substrate.

The drill should be able to fit through the narrow pedicle to prevent damage to the surrounding tissues, resulting in design requirement for the size of the drill. The second lumbar vertebra (L2) has on average the smallest pedicle of the lumbar vertebrae, with an oval cross section of 8.9 mm (SD ± 2.2 mm) by 15 mm (SD ± 1.5 mm) (Zindrick et al., 1987). Manufacturing the two oscillating wheels at this scale is a challenge as the drill diameter is at least twice as large as the wheel diameter. By simplifying the labella motion even further to a single oscillating wheel, the drill can be manufactured with a smaller cross-section and could drill a narrower tunnel (Figure 5C). However, by eliminating a second, opposite rotating wheel, there is a chance that during drilling the lateral forces on the front of the oscillating wheel will result in sideways “walking” of the drill tip. The effect can be limited by making the transmission of the drill rigid in the plane of the wheel rotation to avoid bending of the drill due to the lateral forces, but flexible in the orthogonal plane such that the drill can be used for drilling curved tunnels (Figure 5D).

**FIGURE 6:**
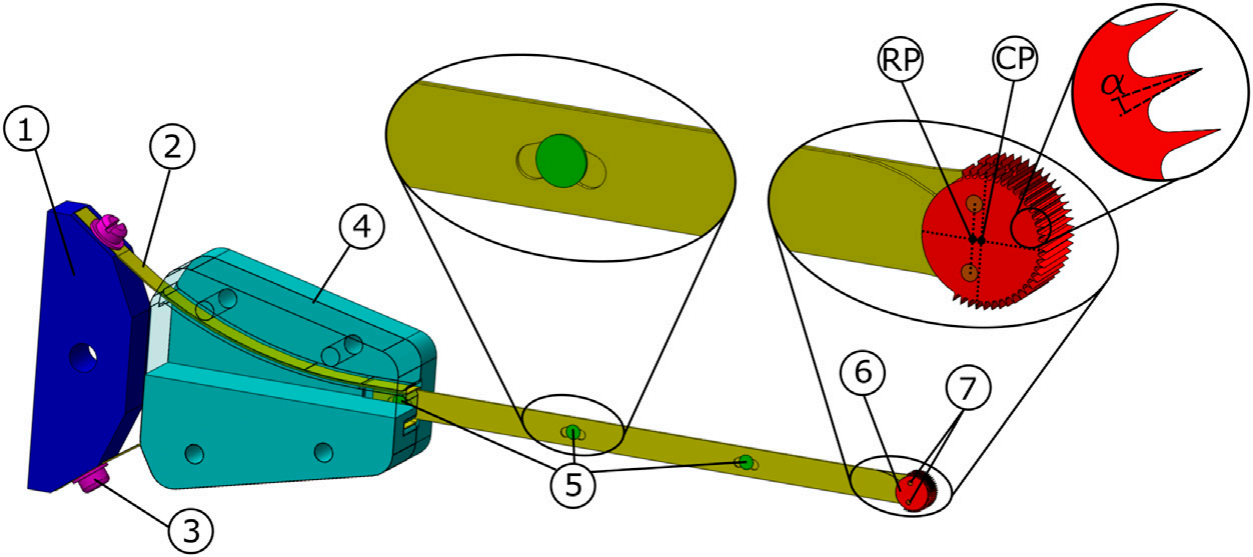
Tsetse Drill design. The actuator (not shown) is connected to an oscillation amplifier 1) to which the two leaf springs 2) are connected via two screws 3). The guide 4) directs both leaf springs, which are interconnected using three transmission pins 5). The transmission pins enable the translation of the leaf springs to activate the drill tip 6). The drill tip is attached to the leaf springs using two drill tip pins 7). The Rotation Point (RP) and the Centre Point (CP) of the drill tip are indicated. The negative rake angle of the teeth is indicated with α .

The oscillatory motion of the drill tip requires an abrasive surface that can cut in both directions. This can be achieved by a cutting surface with symmetrical teeth. The rake angle of the cutting teeth depends, amongst other characteristics, on the material being cut. For bone saws, a negative rake angle of 10° was found most optimal to limit the resultant forces, while still maintaining sawing efficiency (Krause, 1987). Based on this, the drill tip was designed with cutting teeth with a negative rake angle of 10°, a height of 0.5 mm and a distance between the teeth tips of 0.4 mm (Figure 6). As only the frontal surface of the drill tip will be used to cut through the bone, it was decided to cover only this frontal surface with cutting teeth. To ensure effective drilling, an oscillation amplitude of 15° for the drill tip was chosen, as with this amplitude, the travelled distance of one tooth is approximately four times the distance between the teeth. It was decided to cover 220° of the drill tip with teeth so that, even in the most rotated orientation of the drill tip, the frontal area of the drill tip would remain fully covered with teeth.

### 2.3 Transmission design

The transmission must transfer the oscillatory input from the actuator to the drill tip and be flexible in one plane to allow for wall guidance while being rigid in the orthogonal plane to avoid the effect of ‘walking’. This can be achieved by using leaf springs (Figure 6, #2, yellow), as leaf springs can easily bend in one plane, but are rigid in the orthogonal plane. Furthermore, the thickness of the leaf springs can be chosen such that the drill can withstand the axial drilling forces with a reduced risk of buckling. The risk of buckling is limited even further by connecting the two leaf springs at distinct points using transmission pins (Figure 6, #5, green). The transmission pins are rigidly connected to one of the leaf springs, while the other leaf spring is connected via a slot that acts as a sliding joint. This way, the leaf springs are connected while permitting the required translational motion.

The length of the transmission determines the length of the tunnel that can be drilled. In order to drill a straight path along the longitudinal axis of the pedicle from the posterior cortical bone layer to the anterior cortical bone layer is found to be maximum 62 mm (Zindrick et al., 1987). However if the drilled path would follow the cortical bone layer the required tunnel length will be longer. Therefore, the required transmission length should be at least 100 mm.

When retracting the drill, the tip could get stuck when the drill tip (Figure 6, #6, red) is exactly the same size as the tunnel that is cut. To avoid this, the drilled tunnel should be slightly larger than the drill tip. By placing the drill tip pins (Figure 6, #7, orange) that connect the drill tip to the leaf springs slightly closer to the actuator, the drill tip will not rotate around its centre, but will rotate around the centre point between the two pins. As a result, the drill tip will make a slight sweeping motion that ensures that the drilled tunnel is slightly larger than the cross-section of the drill tip. This will ensure that the drill can easily be removed from the drilled tunnel.

### 2.4 Actuator and prototype

**FIGURE 7:**
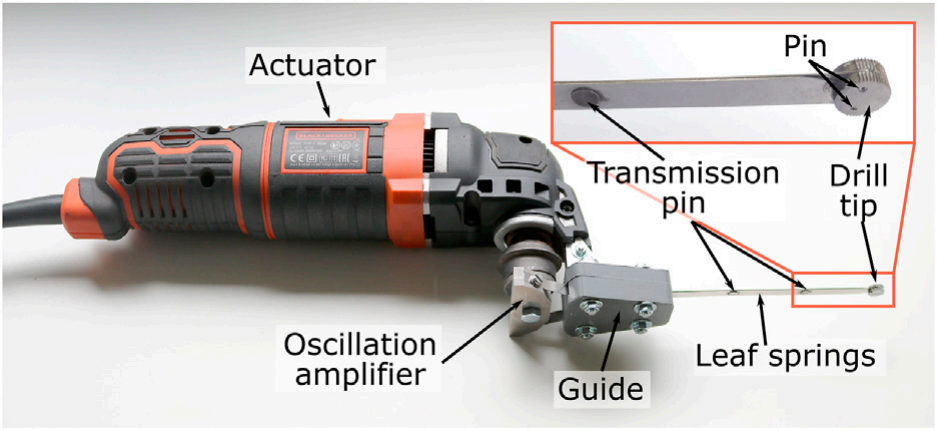
Photograph of the actuator and the Tsetse Drill.

The actuation of the drill tip is achieved using an oscillating input provided by a multi-function tool (Black and Decker MT300KA), see Figure 7. The multi-function tool has an oscillatory output with an oscillation amplitude of 1.4°. The oscillation amplitude of the actuator is increased to 15° amplitude of the drill tip using an oscillation amplifier (Figure 6, #1, blue), that is placed between the actuator and the leaf spring.

The leaf springs (spring steel, thickness 0.3 mm, height 6 mm, length till guide 100 mm, laser cut) are connected to the oscillation amplifier (aluminium, wire EDM and milling), via a guide (Figure 6, #4, turquoise, PolyEthylene Terephthalate Glycol, 3D-printed, Ultimaker 2). The guide has two arc-shaped ridges through which the leaf springs run to prevent buckling of the leaf springs during the actuation of the drill. The leaf springs are connected to each other using three transmission pins (Ø 2 mm, tool-steel, turning lathe). The distal end of each of the leaf springs is connected to the drill tip (hardened steel, wire EDM) using a pin (Ø 1 mm, tool-steel, turning lathe).

## 3 Materials and methods

### 3.1 Experimental goal

The goal of the experiment was two-fold: 1) to evaluate the drilling performance of the prototype through cancellous bone with different bone properties and 2) to investigate the utilisation of wall guidance. Therefore, two different experiments were performed: 1) Drilling Performance Experiment and 2) Wall Guidance Experiment.

### 3.2 Drilling performance experiment

#### 3.2.1 Experimental variables

**Independent Variables**

The following variables were manipulated during the Drilling Performance Experiment.

• Tissue density: In order to determine the Tsetse Drill’s ability to drill through bone with different mechanical properties, bone phantoms with different compressive strengths were used. The experiment was performed in cancellous bone phantom (Sawbones) with a compressive strength of 5, 10 and 15 Pounds per Cubic Foot (PCF), which is comparable to severe osteoporotic, osteoporotic and healthy cancellous bone respectively (Nagaraja and Palepu, 2016).
• Feed-rate: The experiment was conducted with a linear advancement of the drill (feed-rate) of 1 mm/s. This feed rate falls in the range of 0.5–1 mm/s that is commonly used in different bone drilling experiments (Karaca et al., 2011), (Samarasinghe et al., 2021). The experiment on the 15 PCF bone phantom was also conducted with a feed-rate of 0.5 mm/s to investigate whether a lower feed-rate would decrease the axial cutting force.

**Dependent Variables**

The following variables were measured during the Drilling Performance Experiment.

• Axial drilling force: The axial drilling force was measured using a load cell (FUTEK, 25 lbs) that was placed below the bone phantom. Unsuccessful drilling of the drill tip will prevent the drill tip from advancing further into the bone. As a result, the axial drilling force is expected to increase.
• Success rate: The success rate is defined as the percentage of the experiments that could be performed successfully, see Eq. 1. The experiment was considered successful if the full drill path was completed without the need for external intervention.

$$success\;rate=\frac{\#experiments_{successful}}{\#experiments_{total}}∙100\%$$ (1)

#### 3.2.2 Experimental facility

**FIGURE 8:**
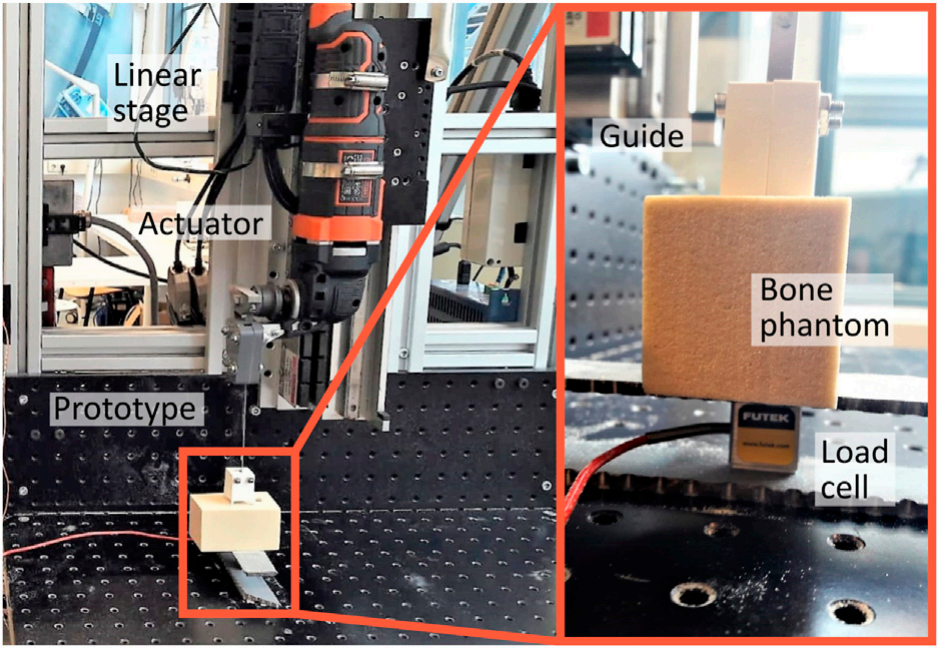
Experimental facility Drilling Performance Experiment. The experimental facility consisted of the prototype of the Tsetse Drill including the actuator connected to the linear stage. The drill tip was guided using a 3D-printed guide. The bone phantom was placed on top of the load cell (Futek, 25 lbs) such that the axial drilling forces could be measured throughout the experiment.

The experimental facility consisted of the prototype with the actuator mounted to a linear stage, which allowed both the feed-rate and the drill depth to be controlled independently (Figure 8). The load cell was placed at the base plate of the linear stage. The bone phantom was placed on top of the load cell such that the axial cutting forces could be measured throughout the experiment. A 3D-printed guide was used to guide the drill tip of the prototype during insertion into the bone phantom.

#### 3.2.3 Experimental protocol

The linear stage was lowered until the drill tip was in close proximity to the bone phantom surface, which was determined by eye. At this time, the guide was placed around the drill. Subsequently, the drill was turned on with a cutting speed of 17,200 RPM, after which the linear stage was turned on and started to move down with the predetermined feed-rate and a drilling depth of 3 cm. The experiment was performed 5 times in the 5, 10 and 15 PCF bone phantoms with a feed-rate of 1 mm/s and 5 times in a 15 PCF bone phantom with a feed-rate of 0.5 mm/s.

#### 3.2.4 Data analysis

The data analysis was performed in MATLAB R2019b. The acquired axial drilling force data were corrected for any offset in the measured force based on the mean measured force before the drilling started. Furthermore, the data were normalised such that the insertion of the drill started at time point t = 0 [s] for each experiment. Statistical analysis was conducted by performing ANOVA analysis to investigate the effect of the compressive strength of the bone phantom on the measured axial drilling force.

### 3.3 Wall guidance experiment

#### 3.3.1 Experimental variables

**Independent variables**

The following variables were manipulated during the Wall Guidance Experiment.

• Insertion angle: The insertion angle was defined as the angle between the drill path and the cortical bone phantom plate. The experiment was conducted with an insertion angle of 5°, 10° and 15°. Successful deflection of the drill for different insertion angles indicates the adaptability and passive steering capabilities of the drill.
• Tissue density: As the use of a steerable bone drill will mainly be of interest in patients with compromised bone due to osteoporosis, the experiment was conducted using 5 PCF rigid foam bone phantoms (Sawbones), which is comparable to osteoporotic bone (Nagaraja and Palepu, 2016). For the 10° insertion angle, the experiment was also conducted using a 10 PCF rigid foam bone phantom (Sawbones), in order to investigate the effect of tissue density on the wall guidance ability.

**Dependent Variables**

The following variables were measured during the Wall Guidance Experiment.

• Success rate: Successful utilisation of wall guidance results drill deflection upon contact with the cortical bone layer. If the deflection is successful, the drill tip continues drilling through the cancellous bone, following the path of least resistance. However, if the deflection is unsuccessful, the drill tip continues its path through the much harder cortical bone layer, resulting in a significantly higher cutting force on the drill and, as a consequence, buckling of the drill. The experiment was considered successful if the full drill path (4 cm) was completed without the need for external intervention to address drill buckling. The success rate is defined as the percentage of the experiments that could be performed successfully, see Eq. 1.
• Drill depth in cortical bone: The effectiveness of using wall guidance will be measured based on the drill depth in the cortical bone 1 cm after initial contact with the cortical bone. The ability to deflect after initial contact with the cortical bone phantom is indicated by the depth of the drill path through the cortical bone. The drill depth in the cortical bone is measured using the depth-measuring blade of a calliper.

#### 3.3.2 Experimental facility

**FIGURE 9:**
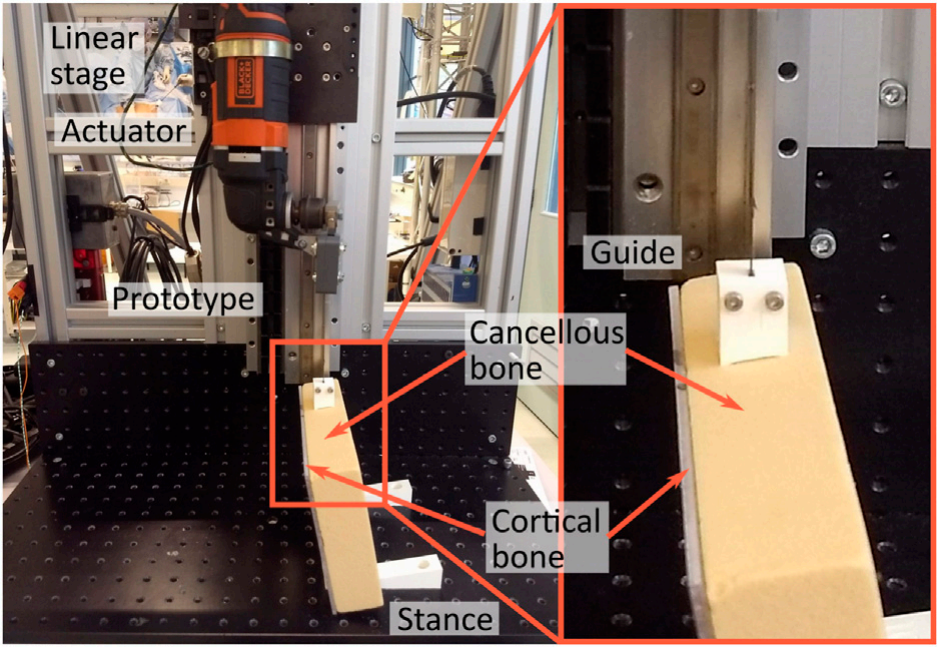
Experimental facility Wall Guidance Experiment. The experimental facility consisted of the prototype of the Tsetse Drill including the actuator connected to the linear stage. The drill tip was guided during insertion into the bone phantom by a 3D-printed guide. The bone phantom consisted of cancellous bone and a layer of cortical bone. The bone phantom was angled (0°, 5°, 10° and 15°) using different stances.

The experimental facility is largely comparable to the experimental facility of the Drilling Performance Experiment and consisted of the Tsetse Drill with the actuator mounted to the same linear stage (Figure 9). The bone phantom consisted of a short fibre epoxy plate (Sawbones) that mimics the cortical bone layer clamped to a rigid foam cancellous bone phantom (Sawbones) of 5 PCF or 10 PCF. The bone phantom was angled using 3D-printed Stances with an angle of 5°, 10° or 15° that were screwed to the baseplate of the linear stage. The drill tip was placed in a guide that was corrected for the angulation of the bone phantom as can be seen in Figure 9.

#### 3.3.3 Experimental protocol

The bone phantom was placed on a stance with a 5°, 10° or 15° angle to affirm the insertion angle of the drill with respect to the cortical bone layer. Subsequently, the Tsetse Drill with the actuator was connected to the linear stage and levelled such that the drill tip with the guide could be fixed to the bone phantom. After this, the linear stage was engaged with a feed-rate of 0.5 mm/s and the drill would start a cycle in which a 4 cm deep tunnel through the bone phantom was followed at a cutting speed of 17,200 RPM. The experiment was repeated until three successful experiments were conducted for each experimental condition.

### 3.4 Data analysis

The data analysis was performed in MATLAB R2019b. An ANOVA analysis was conducted for the statistical analysis of the effect of the insertion angle of the drill and the compressive strength of the bone phantom on the drill depth in the cortical bone.

## 4 Results

### 4.1 Drilling Performance Experiment

**FIGURE 10:**
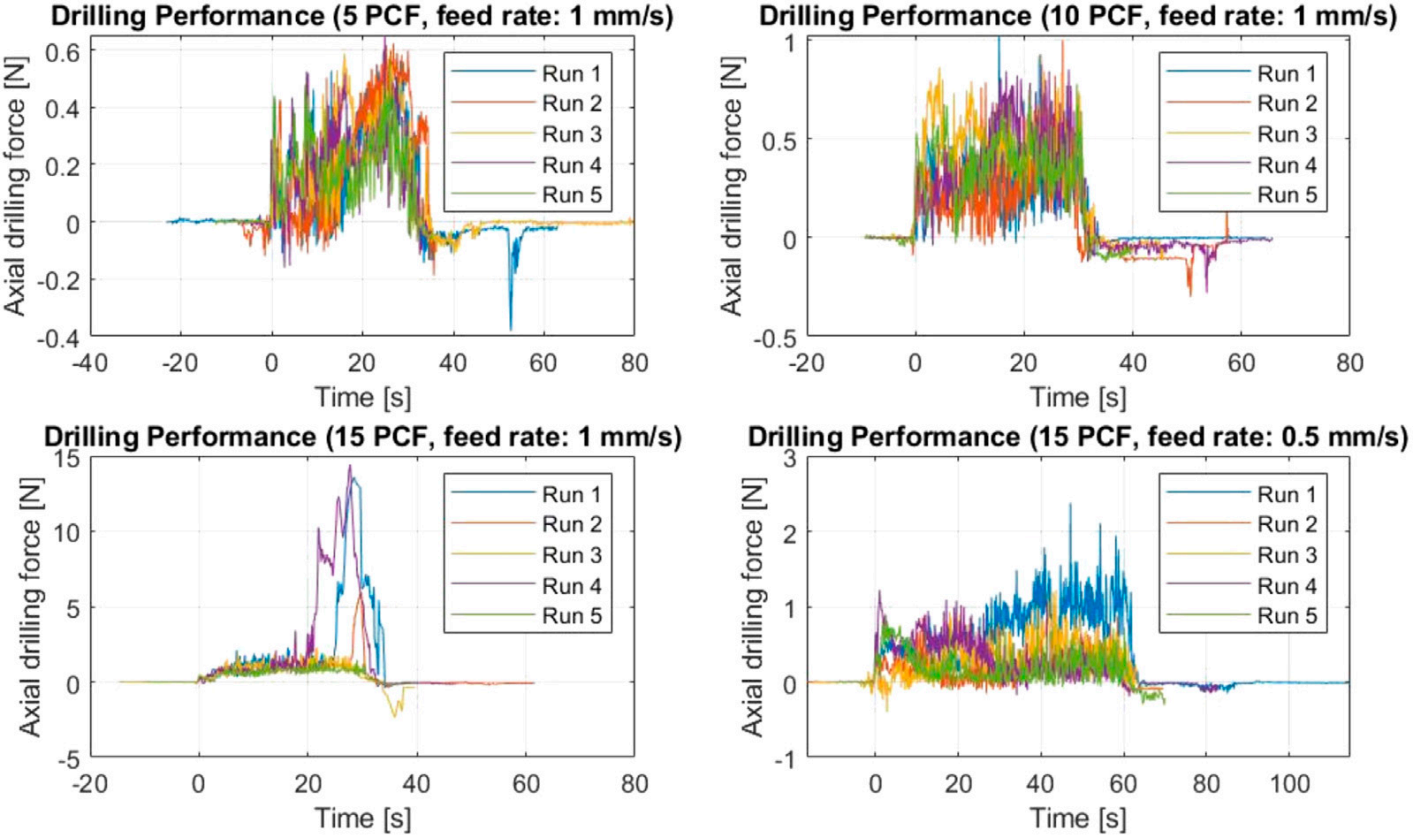
Axial drilling force for each of the experimental conditions of the Drilling Performance Experiment. The data of each run are presented.

Figure 10 shows the axial drilling force *versus* time through the 5, 10 and 15 PCF bone phantom material for the five repetitions of each experimental condition. The success rate was 100% for the 5, and 10 PCF bone phantom with a feed-rate of 1 mm/s. The success rate was also 100% for the 15 PCF bone phantom with a feed-rate of 0.5 mm/s. For the 15 PCF bone phantom with a feed-rate of 1 mm/s the success rate was 80%. The drill buckled during the first run, resulting in the abortion of the experiment. The high axial forces that were measured during Run 2 and Run 4 suggest that the drill was almost buckling during these two runs. However, the experiment was not aborted and thus these two repetitions were considered successful.

**FIGURE 11:**
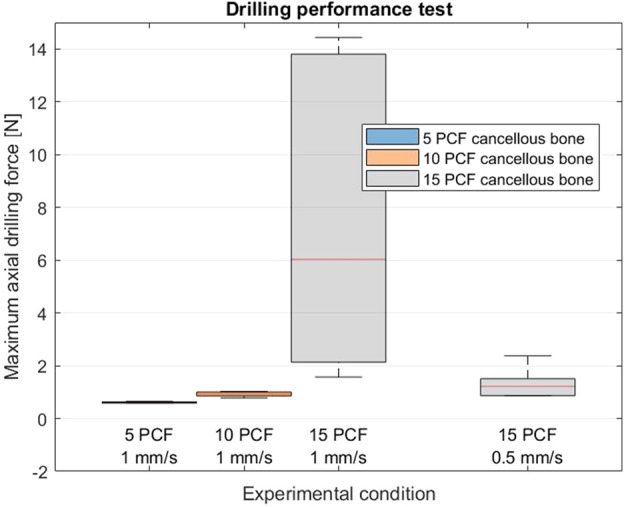
Results of the Drilling Performance Experiment. Boxplots of the maximum axial drilling force measured when drilling through 5, 10 and 15 PCF cancellous bone phantoms at a feed rate of 1 mm/s and for drilling though a 15 PCF cancellous bone phantom at a feed rate of 0.5 mm/s.

Figure 11 shows a boxplot of the maximum axial drilling force for the five repetitions of each experimental condition. The maximum drilling force was 0.61 N (SD ± 0.03 N), 0.92 N (SD ± 0.10 N), 7.59 N (SD ± 6.11 N), for 5 PCF, 10 PCF and 15 PCF bone phantom respectively all with a feed-rate of 1 mm/s. The maximum drilling force was 1.31 N (SD ± 0.62 N) for 15 PCF with a feed-rate of 0.5 mm/s. The buckling of the Tsetse Drill during the first run in the 15 PCF bone phantom with a feed rate of 1 mm/s resulted in a great variance in the maximum axial drilling force measured. The one-way ANOVA test indicated a statistically significant effect of the compressive strength of the bone phantom material on the measured maximum drilling force $\left(p=1.4∙10^{-2}\right)$ . It should be noted that a limited number of data points were used for this statistical analysis.

### 4.2 Wall guidance experiment

**FIGURE 12:**
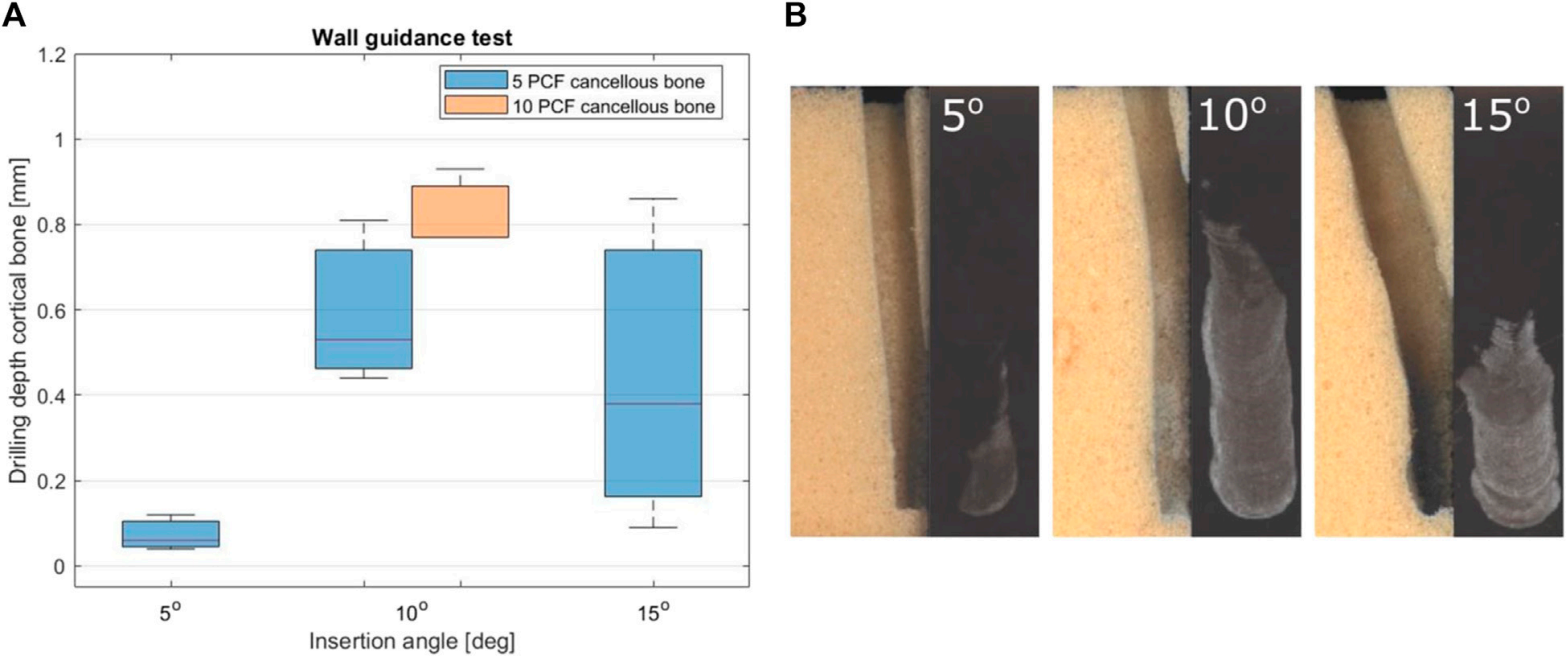
Results of the Wall Guidance Experiment. **(A)** Boxplot of the drilling depth in cortical bone 1 cm after initial contact for three insertional angles (5°, 10° and 15°) in the 5 PCF and 10 CFP cancellous bone phantoms. **(B)** The drill path for three insertion angles (5°, 10° and 15°) in the 5 PCF bone phantom and the damage to the cortical bone phantom.

Figure 12A shows the results of the wall guidance test. The success rate was 100% for the experimental conditions with a 5° and a 10° insertion angle. Figure 12B presents photographs of the drilled tunnels during the wall guidance experiment. The drill buckled once in the experiments with the 15° insertion angle, resulting in a success rate of 75%. This unsuccessful experiment was not included in the data in Figure 12A. The drilling depth in the cortical bone phantom was 0.07 mm (SD ± 0.04 mm), 0.59 mm (SD ± 0.19 mm), 0.44 mm (SD ± 0.39 mm) for an insertion angle of 5°, 10° and 15° all in 5 PCF cancellous bone phantom. The drilling depth in the cortical bone layer with an impact angle of 10° in a 10 PCF cancellous bone phantom was 0.82 mm (SD ± 0.09 mm). The one-way ANOVA test indicated no statistically significant effect of the insertion angle on the drill depth in the cortical bone layer $\left(p=1.0∙10^{-1}\right)$ . Furthermore, the one-way ANOVA test indicated no statistically significant effect of the compressive strength of the cancellous bone on the drill depth in the cortical bone layer $\left(p=1.4∙10^{-1}\right)$ .

## 5 Discussion

### 5.1 Main results

**FIGURE 13:**
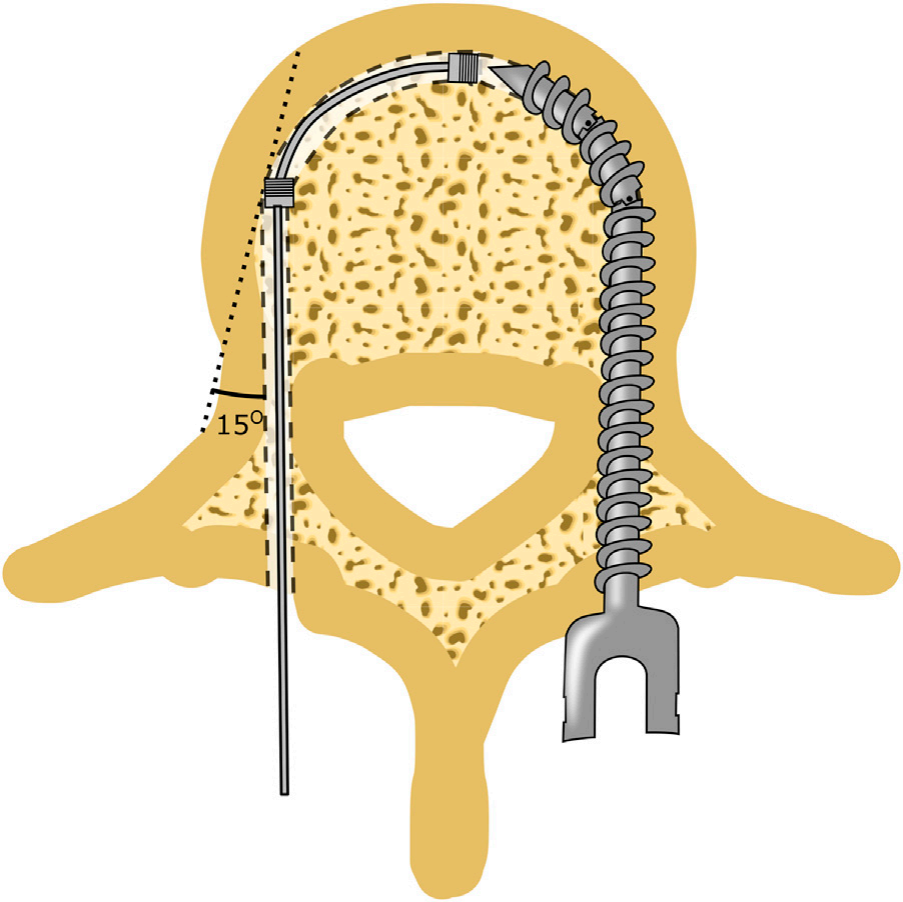
Future vision of using the Tsetse Drill in spinal fusion to increase the fixation strength of spinal bone anchors by increase the contact are between the cortical bone layer and the anchor.

The presented Tsetse Drill (outer dimensions: 4 mm × 7 mm, length: 100 mm) has demonstrated successful drilling through bone phantom material and effective steering along the cortical bone layer using wall-guidance. Figure 13 illustrates a future vision of the Tsetse Drill’s application in spinal fusion surgery. In this scenario, the Tsetse Drill is utilised to create a straight pilot hole through the pedicle. Upon impact with the anterior cortical bone layer, the drill deflects and continues to drill along the cortical bone layer using wall-guidance. After creating this curved tunnel, a spinal bone anchor can be placed, such as a segmented screw capable of bending to follow the curved pre-drilled tunnel. Aghayev *et al.* (Aghayev et al., 2019) and Glerum *et al.* (Glerum et al, 2021) have described these spinal bone anchor designs. The fixation path along the cortical bone layer offers the potential for increased fixation of spinal bone anchors due to the large contact area with the strong cortical bone layer and the macro-shape grip that is achieved through the curved drilling path.

The Tsetse Drill offers a multitude of potential applications beyond spinal fusion surgery, for a range of orthopaedic interventions. For instance, the Tsetse Drill can be utilized in revision surgeries or tendon repairs to access difficult anatomical sites and minimise damage to surrounding anatomy.

The manufactured proof-of-principle prototype of the Tsetse Drill was able to successfully drill through bone tissue phantoms with a compressive strength of 5 PCF, 10 PCF and 15 PCF representing osteoporotic and health cancellous bone. The axial drilling force increased with increasing compressive strength of the bone phantom. One of the five tests performed on the bone phantom with a 15 PCF compressive strength and a feed-rate of 1 mm/s was unsuccessful due to buckling of the drill. Lowering the feed rate can lower the axial drilling force and prevent buckling in bone with higher compressive strength. A feed rate of 0.5 mm/s resulted in a 100% success rate when drilling through 15 PCF cancellous bone phantom. This feed rate is comparable to the feed rate used in other studies (Karaca et al., 2011), (Sezek et al., 2012).

The proof-of-principle prototype was able to successfully drill along the cortical bone phantom when inserted under an angle of 5°, 10° or 15°. For an insertion angle of 5°, the average drill depth in the cortical bone was measured to be 0.07 mm (SD ± 0.04 mm) which is below the average vertebral cortical bone layer thickness of 0.4 mm (Eswaran et al., 2006). However, the drill depth in the cortical bone increased to 0.44 mm (SD ± 0.39 mm) for an insertion angle of 15°. Drilling through the cortical bone is undesired as it could result in a cortical breach and increases the forces acting on the drill. Redesign of the abrasive surface of the drill tip, such as the teeth length, could decrease the cutting depth in the cortical bone layer. Furthermore, it would be interesting to investigate the effect of the drill tip shape on the drilling depth in the cortical bone layer.

An interesting characteristic of the Tsetse Drill is that the drilled tunnel has a rectangular cross-section. Furthermore, the cross-sectional shape of the Tsetse Drill and thus the cross-sectional shape of the drilled tunnel can be changed by redesigning the drill tip. In spinal fusion surgery, a tunnel is created through the pedicle into the vertebral body. Subsequently, a pedicle screw is placed through this tunnel. As of now, the fixation of the pedicle screw mainly relies on the contact between the pedicle screw and the strong cortical bone layer. This contact is, however, limited due to the oval cross-section of the pedicle and the round cross-section of the pedicle screw and tunnel. An oval drill path would allow for novel anchors with an oval cross-section that can shape to the pedicle, such as the one designed by de Kater *et al.* (de Kater et al., 2022). This way the contact area between the anchor and the cortical bone layer in the pedicle could be increased considerably. This would not only result in increased pull-out strength of the anchor, but it can also increase the toggling resistance of the anchor due to the increased contact area with the cortical bone in the caudal and cranial direction.

### 5.2 Limitations and future research

The presented Tsetse Drill was able to successfully deflect when encountering the harder cortical bone with angles up to 15°. The achieved deflection is comparable to the deflection of the drill described by Watanabe *et al.* (Watanabe et al., 2011) without requiring active steering by the user. Furthermore, the drill does not rely on steering cables that require high tension forces to facilitate the required tip deflection. Although this is a large advantage over fully rigid drills, further research is necessary to investigate the ability to utilise wall guidance for larger insertion angles. Additionally, future research could be conducted into combining wall guidance with additional steering methods to increase the adaptability of the drill. Furthermore, the large contact area between the quickly oscillating drill tip and the surrounding bone may result in heat generation. In future research heat generation should be investigated, as heating of bone tissue can result in bone necrosis (Jamil et al., 2020).

For the proof-of-principle experiments, the drill was tested in artificial bone phantom material. Although the mechanical characteristics of this artificial bone are similar to real bone, they are no replacement for actual bone. The pores in the bone phantom are closed cells and smaller than cancellous bone. Furthermore, the pores in cancellous bone are filled with bone marrow, a fatty liquid. The presence of fluids, such as blood and bone marrow, could slow down the disposal of the bone chips that are formed during drilling. Furthermore, the transition zone between the cortical and cancellous bone was not considered in the performed experiments and could influence the wall guidance performance, as the transition from cancellous to cortical bone is less abrupt than in the performed experiments.

The Tsetse Drill is currently designed for spinal fusion surgery. However, the Tsetse Drill could also be of great advantage in ligament reconstructions. During this type of surgery, a ligament is reconstructed by placing a ligament graft through the bone via a pre-drilled tunnel. In these reconstructions, a longer tunnel is thought to increase the fixation of the ligaments (Greis et al., 2001). With the Tsetse Drill, a curved path could be created to increase the tunnel length. Also in other orthopaedic surgeries, such as implant revision surgeries and bone decompression, a steerable drill that can drill along the cortical bone layer could be preferable over conventional bone drills (Alambeigi et al., 2017), (Ahmad Fuad et al., 2018). Therefore, in future research, we will investigate the Tsetse Drill mechanism for use in a variety of orthopaedic interventions.

## 6 Conclusion

This paper presents a novel passively steerable bone drill design inspired by the tsetse fly proboscis. The Tsetse Drill comprises two stacked leaf springs that transmit the oscillation to the drill tip while allowing for bending of the drill. The drill tip of the Tsetse Drill has an abrasive surface and rotates in an oscillatory fashion perpendicular to the drilling direction. This drilling method allows for the utilization of wall guidance to drill a curved path along the cortical bone layer of the vertebra. The proof-of-principle prototype was able to successfully drill through bone phantom material with a compressive strength of 5 PCF, 10 PCF and 15 PCF. Furthermore, the Tsetse Drill was able to passively steer utilizing wall guidance with insertion angles of 5°, 10° and 15°. The presented Tsetse Drill could be a first step in the direction of steerable bone drilling in a variety of orthopaedic procedures, such as spinal fusion surgery.

## Data Availability

The raw data supporting the conclusion of this article will be made available by the authors, without undue reservation.

## References

[B1] Aghayev K. Doulgeris J. J. a Gonzalez B. S. Vrionis F. D. (2019). Transdiscal screw. US10314631B2. https://patents.google.com/patent/US10314631B2/en.

[B2] Ahmad Fuad A. N. B. Deep K. Yao W. Rowe P. (2018). On the development of a new flexible drill for orthopedic surgery and the forces experienced on drilling bovine bone. Proc. Inst. Mech. Eng. H. 232 (5), 502–507. 10.1177/0954411918764508 29543120

[B3] Alambeigi F. Wang Y. Sefati S. Gao C. Murphy R. J. Iordachita I. (2017). A curved-drilling approach in core decompression of the femoral head osteonecrosis using a continuum manipulator. IEEE Robotics Automation Lett. 2 (3), 1480–1487. 10.1109/LRA.2017.2668469

[B4] Bonutti P. (2003). Surgical instrument positioning system. US20030009172A1. Available: https://patents.google.com/patent/US20030009172A1/en .

[B5] Burval D. J. McLain R. F. Milks R. Inceoglu S. (2007). Primary pedicle screw augmentation in osteoporotic lumbar vertebrae: Biomechanical analysis of pedicle fixation strength. Spine 32 (10), 1077–1083. 10.1097/01.brs.0000261566.38422.40 17471088

[B6] Cerkvenik U. Dodou D. van Leeuwen J. L. Gussekloo S. W. S. (2019). Functional principles of steerable multi-element probes in insects. Biol. Rev. 94 (2), 555–574. 10.1111/brv.12467 30259619 PMC7379267

[B7] Cloutier L. P. Aubin C.-E. Grimard G. (2007). Biomechanical study of anterior spinal instrumentation configurations. Eur. Spine J. 16 (7), 1039–1045. 10.1007/s00586-006-0246-1 17205240 PMC2219657

[B8] de Kater E. P. Weststeijn C. F. Sakes A. Breedveld P. “A toggling resistant in-pedicle expandable anchor: A preliminary study,” in Proceedings of the 44th Annual International Conference of the IEEE Engineering in Medicine and Biology Society (EMBC), Glasgow, UK, July. 2022, 3313–3317. 10.1109/EMBC48229.2022.9871068 36086162

[B9] Eswaran S. K. Gupta A. Adams M. F. Keaveny T. M. (2006). Cortical and trabecular load sharing in the human vertebral body. J. Bone Mineral Res. 21 (2), 307–314. 10.1359/jbmr.2006.21.2.307 16418787

[B10] Forsythe B. Collins M. J. Arns T. A. Zuke W. A. Khair M. Verma N. N. (2017). Optimization of anteromedial portal femoral tunnel drilling with flexible and straight reamers in anterior cruciate ligament reconstruction: A cadaveric 3-dimensional computed tomography analysis. Arthrosc. J. Arthrosc. Relat. Surg. 33 (5), 1036–1043. 10.1016/j.arthro.2016.11.004 28117107

[B11] Glerum C. Weiman M. Hessler T. Hill A. Sullivan M. (2021). Pedicle-based intradiscal fixation devices and methods. US2021307924A1. https://patents.google.com/patent/US20210307924A1/en?oq=US2021307924A1.

[B12] Greis P. E. Burks R. T. Bachus K. Luker M. G. (2001). The influence of tendon length and fit on the strength of a tendon-bone tunnel complex: A biomechanical and histologic study in the dog. Am. J. Sports Med. 29 (4), 493–497. 10.1177/03635465010290041901 11476392

[B13] Jamil M. Rafique S. Khan A. M. Hegab H. Mia M. Gupta M. K. (2020). Comprehensive analysis on orthopedic drilling: A state-of-the-art review. Proc. Inst. Mech. Eng. H. 234 (6), 537–561. 10.1177/0954411920911283 32186229

[B14] Jobling B. (1933). A revision of the structure of the head, mouth-part and salivary glands of glossina palpalis rob.-desv. Parasitology 24 (4), 449–490. 10.1017/S0031182000020874

[B15] Karaca F. Aksakal B. Kom M. (2011). Influence of orthopaedic drilling parameters on temperature and histopathology of bovine tibia: An *in vitro* study. Med. Eng. Phys. 33 (10), 1221–1227. 10.1016/j.medengphy.2011.05.013 21703907

[B16] Krause W. R. (1987). Orthogonal bone cutting: Saw design and operating characteristics. J. Biomech. Eng. 109 (3), 263–271. 10.1115/1.3138679 3657116

[B17] Krenn H. W. Aspöck H. (2012). Form, function and evolution of the mouthparts of blood-feeding Arthropoda. Arthropod Struct. Dev. 41 (2), 101–118. 10.1016/j.asd.2011.12.001 22317988

[B18] Lenkbar (2022). FlexMetric-New. Available: https://lenkbar.com/flexmetric-new/ .

[B19] Ma J. H. Sefati S. Taylor R. H. Armand M. (2021). An active steering hand-held robotic system for minimally invasive orthopaedic surgery using a continuum manipulator. IEEE Robotics Automation Lett. 6 (2), 1622–1629. 10.1109/LRA.2021.3059634 PMC8052093 33869745

[B20] McDermott K. W. Liang L. (2006). Overview of operating room procedures during inpatient stays in U.S. Hospitals, 2018: Statistical brief #28. Available: https://www.ncbi.nlm.nih.gov/books/NBK574416 . 34637208

[B21] Nagaraja S. Palepu V. (2016). Comparisons of anterior plate screw pullout strength between polyurethane foams and thoracolumbar cadaveric vertebrae. J. Biomech. Eng. 138 (10). 10.1115/1.4034427 27536905

[B22] Samarasinghe C. Uddin M. Bari S. Xian C. (2021). Temperature and force generation in surgical bone drilling. AIP Conf. Proc. 2324 (1), 060007. 10.1063/5.0037543

[B23] Sendrowicz A. Scali M. Culmone C. Breedveld P. (2019). Surgical drilling of curved holes in bone–a patent review. Expert Rev. Med. devices 16 (4), 287–298. 10.1080/17434440.2019.1596794 30889370

[B24] Sezek S. Aksakal B. Karaca F. (2012). Influence of drill parameters on bone temperature and necrosis: A fem modelling and *in vitro* experiments. Comput. Mater. Sci. 60, 13–18. 10.1016/j.commatsci.2012.03.012

[B25] Stryker (2016). MicroFx brochure.pdf. Available: https://www.stryker.com/content/dam/stryker/sports-medicine/products/microfxosteochondraldrillingsystem/resources/MicroFx%20brochure.pdf .

[B26] Stryker (2019). VersiTomic flexible reamer brochure.pdf. Available: https://www.stryker.com/content/dam/stryker/sports-medicine/products/versitomicflexiblereamingandinterferencescrewsystem/resources/VersiTomic%20flexible%20reamer%20brochure.pdf .

[B27] Wang Y. Zheng H. Taylor R. H. Au K. W. S. (2022). A handheld steerable surgical drill with a novel miniaturized articulated joint module for dexterous confined-space bone work. IEEE Trans. Biomed. Eng. 69, 2926–2934. 10.1109/TBME.2022.3157818 35263248

[B28] Watanabe H. Kanou K. Kobayashi Y. Fujie M. G. “Development of a “steerable drill” for acl reconstruction to create the arbitrary trajectory of a bone tunnel,” in Proceedings of the IEEE/RSJ International Conference on Intelligent Robots and Systems, San Francisco, CA, USA, September. 2011, 955–960. 10.1109/IROS.2011.6094654

[B29] Wu J.-C., (2011). Pedicle screw loosening in dynamic stabilization: Incidence, risk, and outcome in 126 patients. Neurosurg. Focus 31. 10.3171/2011.7.FOCUS11125 21961872

[B30] Zebaze R. Ghasem-Zadeh A. Mbala A. Seeman E. (2013). A new method of segmentation of compact-appearing, transitional and trabecular compartments and quantification of cortical porosity from high resolution peripheral quantitative computed tomographic images. Bone 54 (1), 8–20. 10.1016/j.bone.2013.01.007 23334082

[B31] Zimmerbiomet (2022). Medial portal ACL reconstruction with precision flexible reaming instrumentation - surgical technique. Available: https://www.zimmerbiomet.com/content/dam/zb-corporate/en/products/specialties/sports-medicine/precision-flexible-reaming-system-for-medial-portal-approach/0382.3-GLBL-en%20Precision%20Flexible%20Reaming%20Instrumentation%20SurgTech-Digital.pdf .

[B32] Zindrick M. R. Wiltse L. L. Doornik A. Widell E. H. Knight G. W. Patwardhan A. G. (1987). Analysis of the morphometric characteristics of the thoracic and lumbar pedicles. Spine 12 (2), 160–166. 10.1097/00007632-198703000-00012 3589807

[B33] Zindrick M. R. Wiltse L. L. Widell E. H. Thomas J. C. Holland W. R. Field B. T. (1986). A biomechanical study of intrapeduncular screw fixation in the lumbosacral spine. Clin. Orthop. Relat. Res. 203, 99–112. 10.1097/00003086-198602000-00012 3956001

